# Doped C_20_ fullerenes as a new generation of efficient nanosorbents and nanosensors for rapid detection of dimethyltryptamine in drug detection

**DOI:** 10.1038/s41598-025-30816-6

**Published:** 2025-12-17

**Authors:** Tareq Nafea Alharby, Muteb Alanazi, Jowaher Alanazi

**Affiliations:** 1 Department of Clinical Pharmacy, College of Pharmacy, University of Ha’il, Ha’il, 81442 Saudi Arabia; 2 Department of Pharmacology and Toxicology, College of Pharmacy, University of Ha’il, Ha’il, 81442 Saudi Arabia

**Keywords:** Electrochemical sensor, QTAIM, NCI, Colorimetric sensing, Dimethyltryptamine (DMT), Computational chemistry, Forensic chemistry, Chemistry, Materials science, Nanoscience and technology

## Abstract

The quick and accurate identification of the powerful psychoactive compound Dimethyltryptamine (DMT) is still a significant drawback in forensic science and investigation concerning clinical toxicology. To overcome the issues associated with classical analytical instrumentation, a novel class of innovative highly sensitive nanosensors based on pristine and doped C_20_ fullerenes is presented. Using a heavy computational workflow grounded in Density Functional Theory (DFT) at the computational level B3LYP-D3/6-311G(d, p) in the CPCM solvation model (water phase), we systematically examined the sensing properties of pristine C_20_, and the boron (BC_19_), germanium (GeC_19_), and silicon-doped (SiC_19_) C_20_ fullerenes to DMT. We use calculated values based on theoretical properties relating to the performance (adsorption energy (Eads), HOMO-LUMO gap (HLG), electrical conductivity (σ), and recovery time (τ)). According to the data, each nanomaterial will have its own unique and promising applications. The BC_19_ and SiC_19_ nanostructures presented extremely strong adsorption energies for DMT of -40.78 kcal.mol^− 1^ and − 18.82 kcal.mol^− 1^, respectively, and recovery times that indicated effectively irreversible binding. The combination of high Eads and negligible responsibility change in electrical conductivity of BC_19_ and SiC_19_ suggests they would work well as candidates for adsorption and removal applications where stable analyte capture is desired. On the other hand, the GeC_19_ nanosensor showed an unprecedented and selective response, with adsorption of DMT leading to remarkable increases in the electrical conductivity of the nanomaterial of over 16 orders of magnitude, from 3.4 × 10^− 15^ S.m^− 1^ to 1.9 × 10^2^ S.m^− 1^ while exhibiting a relatively strong adsorption energy of -25.75 kcal.mol^− 1^. This unique alteration identifies GeC_19_ as the top-performing disposable electrochemical sensor for fast, sensitive, and selective detection of DMT. These interactions were also confirmed by NBO, NCI, and QTAIM analyses, which indicated strong charge transfer (NBO), attractive non-covalent interactions (NCI), and medium strength hydrogen bonding (QTAIM) in the BC_19_@DMT, GeC_19_@DMT, and SiC_19_@DMT complexes, respectively. This work not only provides the first theoretical evidence for C_20_-based DMT detection, but also provides a clear pathway for experimental imagining of novel, task-specific nanosensors with important implications for future applications in forensic and point-of-care diagnostics.

## Introduction

The ability to detect DMT is extremely important in a number of fields, including forensic science, clinical toxicology, and regulatory agencies. In forensic science, it is important that DMT can be quickly, and accurately identified in confiscated items or biological specimens, to help law enforcement and court proceedings. Clinically, DMT can have powerful psychoactive effects and a high potential for abuse, therefore will need to be monitored for possible cases of intoxication and overdose and as with any other psychoactive substance reasonable detection methodologies should be used. Due to its powerful psychoactive qualities, DMT has been classified as a Schedule 1 controlled substance in many parts of the world, signifying a high potential for abuse and no accepted medical use. In addition to the aforementioned points, environmental monitoring may require trace detection of DMT in wastewater or public spaces, particularly in areas where DMT use is prevalent^1–4^.

Given its psychoactive properties and legal status, the identification of DMT in forensic, clinical, and environmental samples is of significant importance. Common identification techniques include gas chromatography-mass spectrometry (GC-MS), liquid chromatography-mass spectrometry (LC-MS), high-performance liquid chromatography (HPLC), and Fourier-transform infrared spectroscopy (FTIR). However, these conventional analytical techniques come with several disadvantages: they are often costly, time-consuming, and require trained personnel to operate sophisticated instrumentation. Moreover, their availability is generally limited to well-equipped laboratories, making them impractical for rapid or on-site detection^5–8^.

Due to these limitations, the demand for fast, simple, and accessible detection methods has grown considerably. In recent years, electrochemical and colorimetric sensors have emerged as practical alternatives for real-time and in situ analysis^9,10^. These sensors are increasingly developed using nanomaterials, especially carbon-based nanostructures due to their high surface area, excellent conductivity, and chemical stability^11^. Among the various carbon nanomaterials, carbon nanotubes (CNTs), graphene, and fullerenes are commonly used. Notably, fullerenes have attracted significant attention owing to their symmetric structure, high electron affinity, and versatile surface chemistry, which enhance their interactions with target molecules and make them ideal for sensor applications. Among fullerenes, C_20_, the smallest and highly strained fullerene, has explicitly been explored in several studies for its potential as a chemical sensor^12–15^.

In a recent study, B. Huwaimel et al. highlighted the promising potential of aluminum- and zinc-doped C_20_ fullerenes for the detection of hydrogen sulfide (H_2_S), showcasing their enhanced sensitivity and interaction with toxic gas molecules^16^. Similarly, P. Parkar et al. demonstrated the effective application of lithium-doped C_20_ nanocage as ammonia (NH_3_) gas sensors, emphasizing the tunable electronic properties of doped fullerenes for gas detection^17^. Furthermore, A. Alhowyan et al. proposed C_20_-based nanostructures as highly efficient electrochemical sensors for detecting the psychoactive drug ecstasy, underscoring the versatility of C_20_ derivatives in sensing applications^18^. These studies collectively reinforce the growing interest in C_20_ fullerenes and their doped forms as advanced nanosensors for the detection of hazardous chemicals and illicit substances.

Given the emphasis in various literatures on the sensing potential of fullerene C_20_ and its doped forms, as well as the growing need for rapid and efficient detection of Dimethyltryptamine (DMT), this study focused on evaluating the sensing capability of pristine C_20_ and its boron-, germanium-, and silicon-doped derivatives toward DMT. These elements were chosen due to their distinct electronic characteristics: boron, as an electron-deficient element, can enhance adsorption through charge transfer; germanium offers semiconducting behavior with favorable orbital interactions; and silicon is known for forming strong and stable bonds, which can improve molecular recognition and sensor response^19–22^.

Traditional laboratory methods for synthesizing and testing sensors, although widely used, come with significant drawbacks such as high material costs, time-consuming procedures, the requirement for specialized equipment and expertise, and the potential for experimental errors that can affect reproducibility and precision. To overcome these challenges, we employed computational chemistry techniques, specifically Density Functional Theory (DFT) and the Quantum Theory of Atoms in Molecules (QTAIM)^23–28^. These methods allow for accurate prediction of molecular interactions, electronic properties, and charge transfer mechanisms at the atomic level, while significantly reducing cost and time^29,30^. Moreover, they help minimize experimental uncertainty and guide sensor design before laboratory synthesis.

While there is a pressing demand for rapid and efficient DMT detection, and limitations of current detection methods, this research fills these gaps by showing a novel class of nanomaterial-based sensors utilizing C_20_-doped fullerenes. Our nanosensors are designed for sensitivity, selectivity and real-time detection providing a practical and efficient field-deployable method of detecting DMT in comparison to traditional laboratory-based detection methods. By employing the unique electronic and structural properties of these nanostructures, we intend to meet the growing demand for accessible and rapid DMT detection in both point-of-care and field-based uses. We believe that the results of this work will lead to valuable insights for the future development of efficient, selective and accessible DMT detecting platforms utilizing carbon nanostructures.

## Computational section

**Fig. 1:**
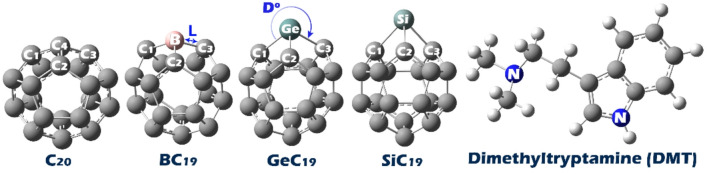
Optimized molecular structures of the pristine C_20_ fullerene, boron- (BC_19_), germanium- (GeC_19_), and silicon-doped (SiC_19_) derivatives, and the Dimethyltryptamine (DMT) molecule. The doped structures were designed to enhance interactions with DMT for sensing applications.

In this study, molecular modeling and structure design were performed in GaussView 6.0, followed by geometric optimization using Gaussian 09 W software (Fig. 1)^31,32^.

**Fig. 2:**
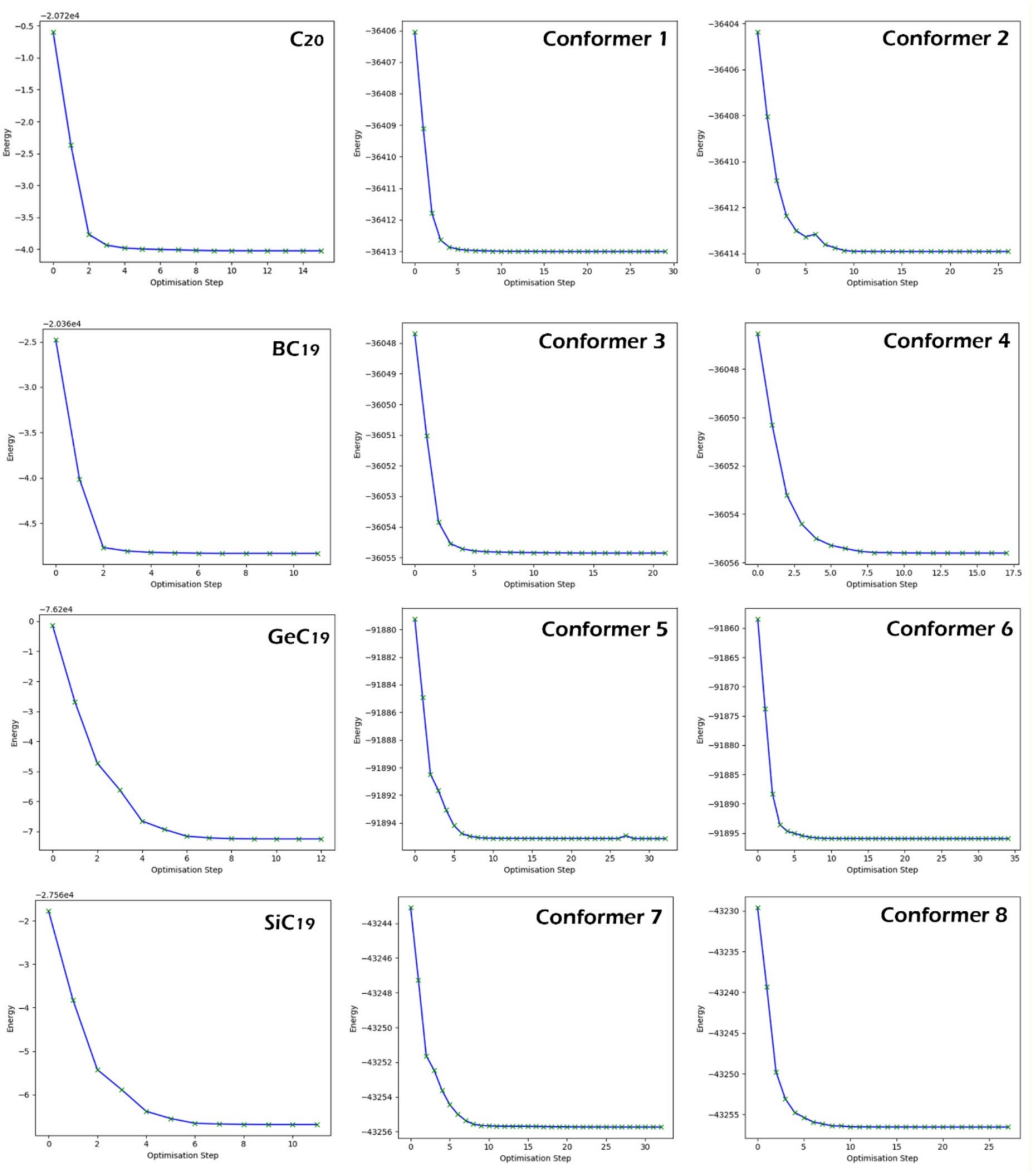
Energy convergence profiles of C_20_, BC_19_, GeC_19_, SiC_19_, and their DMT complexes (Conformers 1–8) showing a rapid decrease and subsequent stabilization of total energy during geometry optimization, confirming attainment of local minima for all structures.

All structures’ geometry optimization convergence was confirmed by observing total energy changes as a function of the optimization step (Fig. 2). In all cases, total energy surged downward at early optimization steps and then stabilized to a constant value, confirming that structures had converged to local minima on the potential energy surface. A lack of residual energy oscillations or fluctuations confirmed numerical stability and indicated that the geometries would be appropriate for the subsequent adsorption energy, recovery time, and electronic conductivity calculations.

The molecular geometries of all studied systems, including DMT, pristine C_20_ fullerene, and its boron-, silicon-, and germanium-doped derivatives (BC_19_, SiC_19_, and GeC_19_), were fully optimized using the B3LYP-D3/6-311G(d, p) level of theory^33,34^. The B3LYP-D3 functional was selected for this work due to its proven reliability in predicting the structural and electronic properties of nanomaterials and organic molecules^35,36^. All calculations were performed in the water phase with the Conductor-like Polarizable Continuum Model (CPCM)^37^. Water was chosen as the solvent in the CPCM model to represent polar environments where DMT is typically found such as biological fluids (i.e., blood, urine, plasma) and environmental samples (i.e., wastewater). These water systems play a key role in forensic and ecological investigations involving psychoactive substances. Thus, simulating in water accounts for correctly solvation effects, hydrogen bonding, and dielectric screening, all of which greatly influence the adsorption of DMT and overall sensor performance. On the other hand, a gas-phase or organic-phase model would significantly over- or underestimate these effects. Hence, the water media is the most appropriate simulation in representing the condition of DMT detection in real life. In addition to geometric optimizations, the UV-Vis adsorption spectra of all designed structures were calculated using the time-dependent density functional theory (TD-DFT) method^38,39^. In these calculations, the number of excited states was set to nstates = 20 to ensure inclusion of all low-lying singlet excitations contributing to the visible region. This number was determined after convergence testing, confirming that the primary adsorption peaks were fully captured without introducing spurious higher-energy transitions. The same theoretical level and solvation model were used for all pristine and adsorbed systems to maintain consistency. Also, frequency calculations were performed at the same theoretical level and no imaginary frequencies were observed, indicating that all optimized geometries are stable and not in a transient state.

The most important parameters for investigating structural and electronic properties, including cohesive energy and energy gap (HLG), chemical softness (S), chemical hardness (η), chemical potential (µ), maximum charge transfer value (ΔNmax), and electrophilicity-based charge transfer (ECT), were calculated using Eq. 1 to 7, respectively^40–45^.

1 $$\:{\varvec{E}}_{\varvec{C}\varvec{o}\varvec{h}}=-({\varvec{E}}_{\varvec{t}\varvec{o}\varvec{t}\varvec{a}\varvec{l}}-\sum\nolimits_{\varvec{i}}{\varvec{n}}_{\varvec{i}}{\varvec{E}}_{\varvec{i}})/\varvec{n}.$$

2 $$\:\mathbf{H}\mathbf{L}\mathbf{G}=\left|{\mathbf{E}}_{\mathbf{H}\mathbf{O}\mathbf{M}\mathbf{O}}-{\mathbf{E}}_{\mathbf{L}\mathbf{U}\mathbf{M}\mathbf{O}}\right|$$

3 $$\:\varvec{\upeta\:}=\raisebox{1ex}{$(-{\mathbf{E}}_{\mathbf{H}\mathbf{O}\mathbf{M}\mathbf{O}}-(-{\mathbf{E}}_{\mathbf{L}\mathbf{U}\mathbf{M}\mathbf{O}}\:\left)\right)$}\!\left/\:\!\raisebox{-1ex}{$2$}\right.$$

4 $$\:\varvec{\upmu\:}=-(-{\mathbf{E}}_{\mathbf{H}\mathbf{O}\mathbf{M}\mathbf{O}}+(-{\mathbf{E}}_{\mathbf{L}\mathbf{U}\mathbf{M}\mathbf{O}}\left)\right)/2$$

5 $$\:\varvec{S}=1/2\varvec{\upeta\:}$$

6 $$\:{\varDelta\:\varvec{N}}_{\varvec{m}\varvec{a}\varvec{x}}=-\raisebox{1ex}{$\varvec{\mu\:}$}\!\left/\:\!\raisebox{-1ex}{$\varvec{\eta\:}$}\right.$$

7 $$\:\varvec{E}\varvec{C}\varvec{T}={\left({\varvec{\Delta\:}\varvec{N}}_{\varvec{m}\varvec{a}\varvec{x}}\right)}_{\varvec{\alpha\:}}-{\left({\varvec{\Delta\:}\varvec{N}}_{\varvec{m}\varvec{a}\varvec{x}}\right)}_{\varvec{\beta\:}}$$

In Eq. 1: The total electronic energy of the optimized molecule or complex is denoted by Etotal. The summation of the total energies of the isolated atoms forming the structure is represented by ∑, where is the energy of the atom and is the number of atoms of that type in the structure. is the total number of atoms in the structure. Also, the energies of the highest occupied molecular orbital (HOMO) and the lowest unoccupied molecular orbital (LUMO) are represented by E_HOMO_ and E_LUMO_, respectively (In Eqs. 2-4).

In Eqs. 6 and 7: ΔNmax represents the maximum charge transfer. A positive ECT indicates that the sensor donates electrons to the DMT, while a negative ECT suggests that electron transfer occurs from the DMT to the sensor (α refers to ΔNmax for the complex and β refers to ΔNmax for C_20_ doped with (B/Si/Ge))^46,47^.

The sensing properties were calculated and investigated using Eqs. 8–10.

8 $$\:{\mathbf{E}}_{\mathbf{a}\mathbf{d}\mathbf{s}}={\mathbf{E}}_{\left(\mathbf{R}-\right)\mathbf{C}20@\mathbf{D}\mathbf{M}\mathbf{T}}-\left({\mathbf{E}}_{\varvec{D}\varvec{M}\varvec{T}}+{\mathbf{E}}_{\left(\mathbf{R}-\right)\mathbf{C}20}\right)+{\mathbf{E}}_{\mathbf{B}\mathbf{S}\mathbf{S}\mathbf{E}}$$

9 $$\:\varvec{\tau\:}={\varvec{V}}_{0}^{-1}\times\:\mathbf{e}\mathbf{x}\mathbf{p}(-\frac{{\varvec{E}}_{\varvec{a}\varvec{d}\varvec{s}}}{{\varvec{k}}_{\varvec{B}}\varvec{T}})$$

10 $$\:\varvec{\upsigma\:}=\varvec{A}{\varvec{T}}^{3/2}{\varvec{e}}^{(-\varvec{H}\varvec{L}\varvec{G}/2\varvec{K}\varvec{T})}$$

In Eq. (8), the adsorption energy (Eads) is computed using the total energy of the DMT–sensor complex (E(R-)C_20_@DMT), the energies of the isolated DMT (E_DMT_) and the sensor (E(R-)C_20_). Also, to accurately quantify intermolecular interactions, the basis set superposition error (BSSE) was systematically corrected using the counterpoise method during adsorption energy (Eads) calculations. This approach ensures that artificial stabilization due to basis set incompleteness is minimized, leading to more reliable interaction energies. Equation (9) defines the recovery time (τ) based on the adsorption energy, where V_0_ is the attempt frequency (typically ~ 10^12^ s^− 1^), k_B_ is the Boltzmann constant, and T is the temperature (298 K). Lastly, Eq. (10) describes the electrical conductivity (σ), where A is the Richardson constant (6 × 10⁵ A.m^− 2^.K^− 2^), and T is the temperature (298 K)^48–53^.

Also, the dipole moment was calculated using Eq. 11^54^.

11 $$\:\varvec{\mu\:}=\sqrt{{\varvec{\mu\:}}_{\varvec{x}}^{2}+{\varvec{\mu\:}}_{\varvec{y}}^{2}+{\varvec{\mu\:}}_{\varvec{z}}^{2}}$$

These calculations were meticulously carried out to explore the adsorption characteristics, electronic properties, and sensing capabilities of C_20_ fullerene and its functionalized derivatives for DMT detection, aiming to advance the development of diagnostic technologies.

## Results and discussion

### Structural properties

#### Bond length and bond angle

When designing molecules, bond length and bond angle are crucial factors as they determine the overall geometry and stability of the molecule. The bond length affects the strength of the bond, with shorter bonds typically being stronger, while bond angles influence the spatial arrangement and reactivity of the molecule. Introducing a new atom into a molecule can alter these parameters by changing electron distribution, which in turn affects the bond lengths and angles. Doping atoms into a molecule, particularly in organic semiconductors or materials science, can have a significant effect on the mobility of π-electrons. The dopants can introduce new energy levels, disrupt conjugation, or create localized states within the electronic structure. This often results in a change in the electronic band structure, which can either increase or decrease the mobility of π-electrons depending on the type and concentration of the dopant^55–58^. Based on these explanations, the bond length and bond angle were investigated for each of the designed structures, and the results are reported in Table 1.

**Table 1:** Calculated values ​​of bond lengths (L) and bond angles (D) between some important atoms in the designed structures.

Structure	Bond lengths (Å)		Bond angles (°)	
C_20_	C4-C1	1.44	C1-C4-C2	108.65
	C4-C2	1.44	C1-C4-C3	108.99
	C4-C3	1.44	C2-C4-C3	108.65
BC_19_	B-C1	1.56	C1-B-C2	108.28
	B-C2	1.55	C1-B-C3	108.29
	B-C3	1.55	C2-B-C3	104.60
GeC_19_	Ge-C1	2.10	C1-Ge-C2	75.10
	Ge-C2	2.10	C1-Ge-C3	75.08
	Ge-C3	2.10	C2-Ge-C4	75.08
SiC_19_	Si-C1	1.99	C1-Si-C2	77.87
	Si-C2	1.99	C1-Si-C3	77.86
	Si-C3	1.99	C2-Si-C3	77.85

For the C_20_ structure, which consists of carbon atoms, the bond lengths between atoms (C4-C1, C4-C2, and C4-C3) are all 1.44 Å, typical for carbon-carbon bonds in a sp^2^ hybridized structure. The bond angles between the carbon atoms (C1-C4-C2, C1-C4-C3, and C2-C4-C3) are very close to each other, varying between 108.65° and 108.99°, indicating a nearly ideal tetrahedral geometry typical of sp^2^ hybridization. This suggests that the molecule maintains a balanced, stable structure with minimal strain.

In the BC_19_ structure, introducing boron leads to an increase in bond lengths. The B-C1 bond length is 1.56 Å, which is slightly longer than the typical C-C bond length observed in C20, reflecting the larger atomic radius of boron. The bond angles in BC19 (C1-B-C2, C1-B-C3) are relatively close to each other (around 108.28° to 108.29°), but there is a noticeable decrease in the C2-B-C3 bond angle (104.60°). This suggests a slight distortion in the structure due to the presence of boron, likely because of its electron-deficient nature, which creates some bond angle strain.

In the GeC_19_ structure, where germanium replaces carbon, the bond lengths (Ge-C1, Ge-C2, and Ge-C3) are significantly longer at 2.10 Å. Germanium atoms are larger than carbon atoms, and this larger size leads to elongated bonds. The bond angles in GeC19 (C1-Ge-C2, C1-Ge-C3, and C2-Ge-C4) are very small, ranging from 75.08° to 75.10°, much smaller than those seen in the carbon-based structures. This substantial reduction in bond angles indicates considerable strain in the molecule, likely due to the larger atomic size of germanium and the forced bonding geometry to accommodate the larger atoms.

The SiC19 structure, with silicon replacing carbon, shows bond lengths of 1.99 Å (Si-C1, Si-C2, and Si-C3), which are also longer than the bond lengths in C_20_ but shorter than those in GeC_19_. Silicon atoms are intermediate in size between carbon and germanium, which is reflected in the bond lengths. The bond angles (C1-Si-C2, C1-Si-C3, and C2-Si-C3) are all relatively small, between 77.85° and 77.87°, much smaller than those in C_20_ and BC_19_, suggesting that the molecule experiences significant strain due to the larger atomic size of silicon and the resulting tight bonding geometry.

#### Cohesive energy

**Fig. 3:**
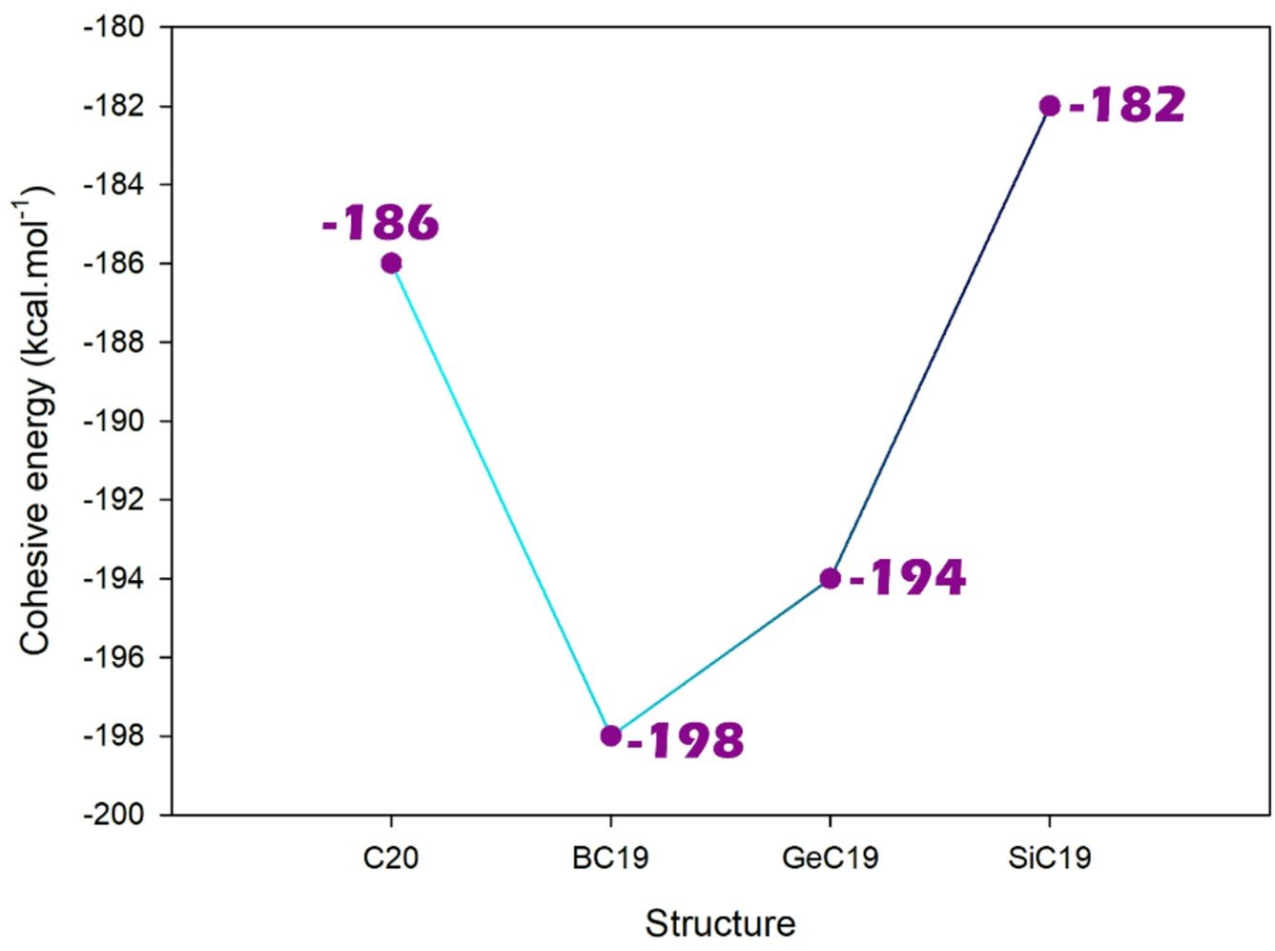
Variation of cohesive energy (in kcal.mol^− 1^) for different nanocluster structures (C_20_, BC_19_, GeC_19_, and SiC_19_). The BC_19_ structure exhibits the highest cohesive energy (The most negative) (−198 kcal.mol^− 1^), indicating the highest stability among the compared clusters.

Cohesive energy refers to the amount of energy required to break apart a material into its constituent atoms or molecules. It reflects the strength of the interactions that hold the material together. In the context of doping, introducing foreign atoms or molecules can alter the cohesive energy of the base material^59,60^. On this basis, the cohesive energy for pristine C_20_ fullerene and its doped forms was computationally studied, and the results are reported in Fig. 3.

The pristine C_20_ structure exhibits a cohesive energy of −186 kcal.mol^− 1^, serving as the reference for comparison. Upon boron doping, the cohesive energy significantly increases to −198 kcal.mol^− 1^ in the BC_19_ structure, indicating that boron incorporation substantially enhances the structural stability of the fullerene, likely due to stronger B-C bonding interactions. GeC_19_ also shows an improvement in cohesive energy, reaching − 194 kcal.mol^− 1^, suggesting that germanium doping contributes positively to the overall cohesion of the structure, potentially through favorable electronic compatibility and atomic size matching with carbon. In contrast, SiC_19_ presents the lowest cohesive energy value at −182 kcal.mol^− 1^, implying that silicon doping reduces the stability of the fullerene framework, possibly due to weaker Si-C bonds and structural strain from size mismatch. Based on these results, boron appears as the most effective impurity for increasing stability.

The observed cohesive energy trends are well supported by the bond geometry data (bond length and bond angle). BC_19_ has more regular bond angles and shorter, stronger bonds correspond to higher cohesive energy and thus greater stability. In contrast, the longer bond lengths and highly distorted angles in SiC_19_ and GeC_19_ indicate localized strain and weaker bonding, which is associated with reduced structural stability.

### Electronic properties

#### MEP contours

**Fig. 4:**
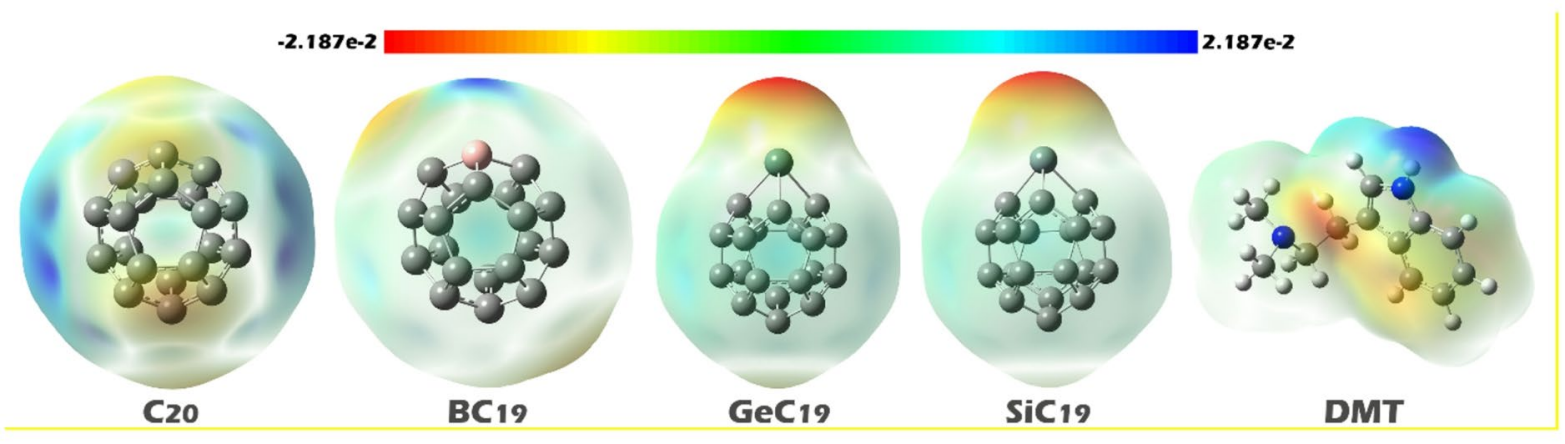
Molecular electrostatic potential (MEP) maps of C_20_, BC_19_, GeC_19_, SiC_19_, and DMT molecules. The color scale ranges from − 2.187 × 10^− 2^ to 2.187 × 10^− 2^, indicating regions of electron-rich (red) and electron-deficient (blue) areas, respectively. These maps illustrate the charge distribution and potential reactive sites of each structure.

Molecular Electrostatic Potential (MEP) contours play a crucial role in identifying the most likely interaction sites in molecules by visualizing the distribution of electrostatic potential across the molecular surface^61^. Typically, in MEP maps, red regions represent areas of high electron density and thus negative electrostatic potential, often corresponding to electronegative atoms; these are the most likely sites for electrophilic attack or hydrogen bonding with positively charged or electron-deficient species^62^. On the other hand, blue regions signify low electron density or positive electrostatic potential, usually located around hydrogen atoms or electron-deficient centers, and are favorable sites for nucleophilic attack or interactions with electron-rich groups^63^. Green regions represent neutral potential and are typically less reactive. Analysis of these colored lines allows the identification of the most likely regions for intermolecular interactions, which is essential for the design of complexes, especially in applications such as molecular diagnostics, drug receptor binding, and sensor development^64,65^. Based on these explanations, the MEP contours for each of the designed structures and also for the DMT were shown in Fig. 4.

In the MEP contour of the DMT molecule, two distinct electrostatic regions are observed: the blue region is located on the nitrogen atom of the indole ring, indicating an electron-deficient site, while the red area is centered on the methylated nitrogen atom, showing it to be electron-rich. These two nitrogen atoms represent chemically different active sites, each capable of forming interactions with different types of surfaces or dopant atoms, and each contributing unique properties to any resulting complex.

In the case of pristine C_20_, the MEP contour shows that the blue color is diffusely spread across the surface, suggesting a relatively uniform electron-deficient character around the molecule. As such, C_20_ is not strongly polarized and may engage in weak electrostatic or dispersion interactions, primarily with the electron-rich methylated nitrogen atom of DMT (the red region). However, the lack of a well-defined active site in C_20_ means that its interaction with DMT may be less selective and structurally flexible, leading to relatively weak binding and less impact on the electronic structure of the complex.

In BC_20_, the blue color is highly localized on the boron atom, marking it as a strongly electron-deficient center. This makes it a favorable site for interaction with the methylated nitrogen atom of DMT, which has high electron density. The strong electrostatic attraction between these two regions can lead to a stable and well-oriented complex, where charge transfer or dipole alignment may significantly influence the electronic properties of the system.

In contrast, GeC_20_ and SiC_20_ exhibit red regions on the Ge and Si atoms, respectively, indicating that these dopant atoms are electron-rich. This makes them well suited to interact with the indole nitrogen atom of DMT (the blue region), which is electron-deficient.

**Fig. 5:**
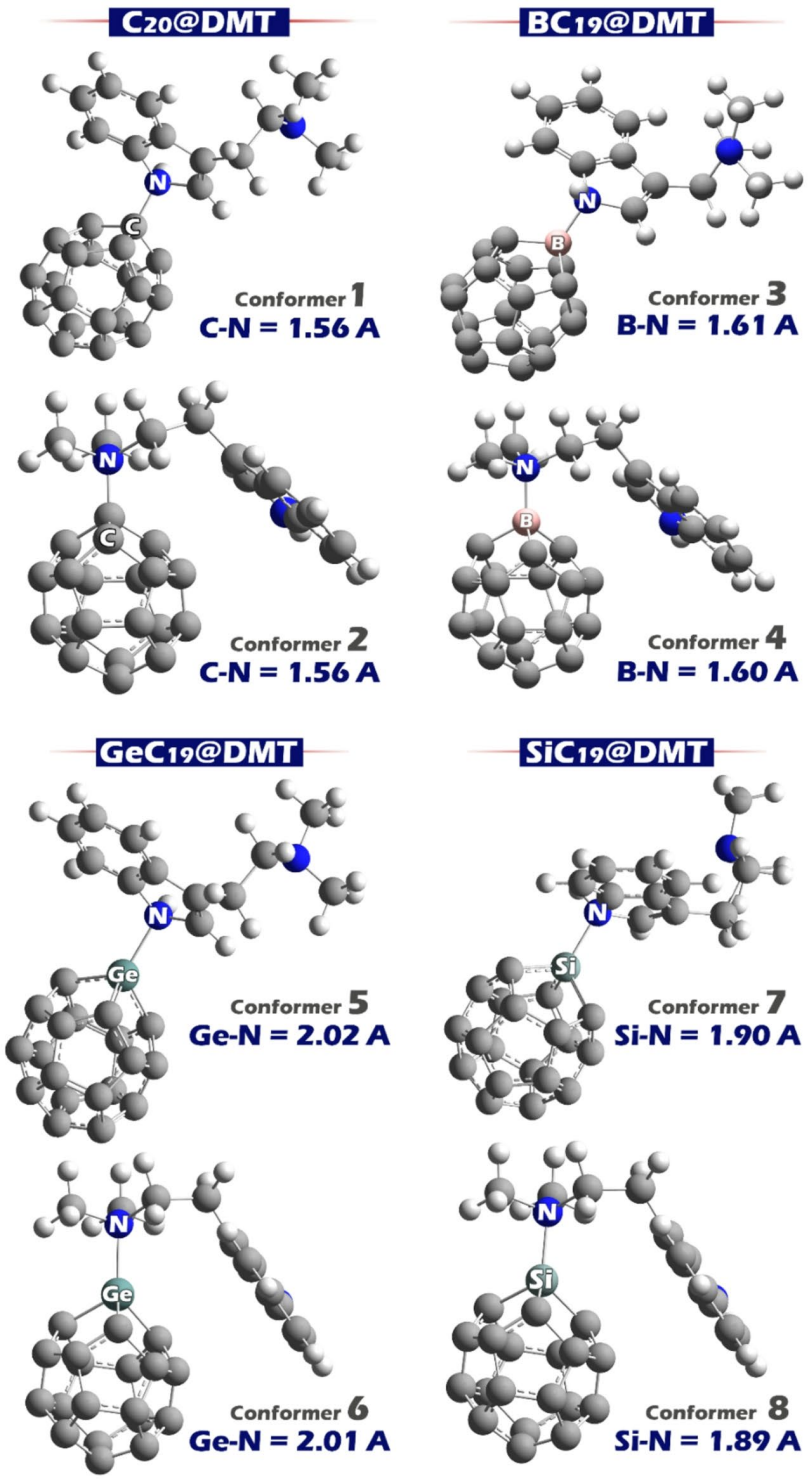
Optimized geometries of DMT complexes with C_20_, BC_19_, GeC_19_, and SiC_19_ clusters (denoted as C_20_@DMT, BC_19_@DMT, GeC_19_@DMT, and SiC_19_@DMT). Each system shows two conformers with corresponding bond lengths between the dopant atom and nitrogen (C-N, B-N, Ge-N, and Si-N). The bond distances range from 1.56 Å to 2.02 Å, reflecting variations in interaction strength across the different sensor-DMT complexes.

Based on these results, two conformational forms were considered for each of the designed complexes (via the two active sites of DMT). The optimized structure of each of the designed conformers is shown in Fig. 5.

In the MEP contour of the DMT molecule, two distinct electrostatic regions are observed: the blue region is located on the nitrogen atom of the indole ring, indicating an electron-deficient site, while the red area is centered on the methylated nitrogen atom, showing it to be electron-rich. These two nitrogen atoms represent chemically different active sites, each capable of forming interactions with different types of surfaces or dopant atoms, and each contributing unique properties to any resulting complex. For C_20_@DMT (Conformers 1 and 2), the C-N bond lengths are 1.566 Å and 1.563 Å, respectively.

In BC_19_@DMT (Conformers 3 and 4), the B–N bond lengths measure 1.617 Å and 1.607 Å, which are longer than the C-N bonds but still relatively short, indicating strong electrostatic interactions. The boron atom in BC₁₉ is highly electron-deficient (blue region in MEP), strongly attracting the electron-rich methylated nitrogen in DMT. The shorter B-N bond in conformer 4 (1.607 Å) likely reflects a more favorable orientation and interaction, indicating enhanced stability and binding. For GeC_20_@DMT (Conformers 5 and 6), the Ge-N bond lengths are notably longer at 2.028 Å and 2.015 Å, representing the weakest interactions among the complexes.

In SiC_19_@DMT (Conformers 7 and 8), Si-N bond lengths of 1.900 Å and 1.893 Å are observed. These are shorter than the Ge-N bonds. Similar to GeC_19_, the Si atom is electron-rich (red in MEP) and interacts with the indole nitrogen (blue). The slightly shorter bond in conformer 8 represents a more favorable binding geometry and is expected to have a stronger interaction compared to conformer 7.

#### Reactivity parameters

The key reactivity parameters of chemical systems include the energy gap (HLG), chemical softness (S), chemical hardness (η), chemical potential (µ), maximum charge transfer (ΔNmax), and electrophilicity-based charge transfer (ECT). The HLG determines the stability and reactivity, with a smaller gap indicating higher reactivity^43,66^. Chemical softness reflects a molecule’s ability to accept or donate electrons, with softer molecules being more reactive, while chemical hardness measures resistance to electron density changes, with harder molecules being less reactive^41^. The chemical potential indicates a molecule’s tendency to gain or lose electrons, guiding electron flow during reactions^67^. Maximum charge transfer quantifies the extent of electron movement between donor and acceptor molecules, which is important for redox reactions^68^. Finally, electrophilicity-based charge transfer gauges a molecule’s tendency to accept electrons^69^. Accordingly, each of these parameters was subjected to computational study, and the results were reported in Table 2.

**Table 2:** Energy values ​​of orbitals HOMO (eV) and LUMO (eV) as well as HLG (eV), η (eV), S (eV^− 1^), µ (eV), ∆N_max_ and ECT for each of the conformers studied in this work.

Structure	Conformer	LUMO	HOMO	HLG	η	µ	S	∆*N*_max_	ECT
Sensor									
C_20_	**-**	−3.42	−5.37	1.95	0.97	−4.39	0.51	4.5	**-**
BC_19_	**-**	−3.43	−5.34	1.91	0.95	−4.38	0.52	4.6	**-**
GeC_19_	**-**	−3.45	−6.17	2.72	1.36	−4.81	0.36	3.5	**-**
SiC_19_	**-**	−3.5	−5.87	2.37	1.18	−4.68	0.42	3.9	**-**
Complex/Conformer									
C_20_@DMT	1	−2.7	−4.62	1.92	0.96	−3.66	0.52	3.8	0.69
	2	−2.58	−4.52	1.94	0.97	−3.55	0.51	3.6	0.84
BC_19_@DMT	3	−2.77	−4.68	1.91	0.95	−3.72	0.52	3.9	0.69
	4	−2.64	−4.55	1.91	0.95	−3.59	0.52	3.7	0.82
GeC_19_@DMT	5	−2.74	−3.59	0.85	0.42	−3.16	1.17	7.4	−3.91
	6	−2.7	−4.52	1.82	0.91	−3.61	0.54	3.9	−0.43
SiC_19_@DMT	7	−2.79	−4.65	1.86	0.93	−3.72	0.53	4.1	−0.04
	8	−2.69	−4.55	1.86	0.93	−3.62	0.53	3.9	0.06

The data in Table 2 first outlines the electronic properties of the pristine sensors C_20_, BC_19_, GeC_19_, and SiC_19_ before interaction with DMT. Among these, C_20_ and BC_19_ have similar HOMO and LUMO energies, with HOMO levels around − 5.34 to −5.37 eV and LUMO levels near − 3.42 to −3.43 eV, resulting in small HOMO-LUMO gaps (HLG) of approximately 1.91–1.95 eV. This suggests a moderate electronic stability and reactivity balance. GeC_19_ and SiC_19_ show larger HLG values of 2.72 eV and 2.37 eV, respectively, with lower HOMO energies (especially GeC_19_ at −6.17 eV) and slightly more negative chemical potentials (µ), indicating increased chemical hardness and reduced softness compared to C_20_ and BC_19_.

Upon complex formation with DMT, notable changes in electronic properties are observed. For both C_20_@DMT and BC_19_@DMT conformers, the HOMO and LUMO energies shift upward (HOMO becomes less negative), but the HLG remains essentially unchanged around 1.91–1.94 eV. Chemical hardness (η) and softness (S) values are largely stable, with slight increases in chemical potential (µ) indicating some electron density redistribution. The ∆Nmax decreases moderately, reflecting altered electronic interactions. Importantly, the ECT values for these complexes are positive (0.69–0.84), which suggests that charge transfer occurs from the sensor to the DMT molecule.

In contrast, the GeC_19_@DMT complexes exhibit significant changes. Conformer 5 shows a dramatic decrease in the HLG to 0.85 eV from 2.72 eV in the pristine sensor, accompanied by a large reduction in chemical hardness (0.42 from 1.36) and a substantial increase in softness (1.17 compared to 0.36). The ∆Nmax increases markedly to 7.44, indicating enhanced electronic reactivity. Furthermore, the ECT value is strongly negative (−3.91), indicating electron transfer from DMT to the sensor. Conformer 6, however, displays a smaller change with an HLG of 1.82 eV and an ECT closer to zero (−0.43), implying less pronounced charge transfer and moderate changes in electronic properties compared to the free GeC_19_.

For SiC_19_@DMT complexes, the HOMO-LUMO gaps slightly decrease from 2.37 eV in the pristine sensor to about 1.86 eV in both conformers 7 and 8. Chemical hardness decreases modestly, while softness increases slightly, indicating somewhat enhanced reactivity upon DMT binding. The maximum charge transfer values remain near 4, similar to the pristine sensor. The ECT values for both conformers are − 0.04 and − 0.06, respectively, indicating electron transfer from DMT to SiC_19_.

According to the obtained results, while the C_20_ and BC_19_ sensors experience small changes in electronic parameters and electron flow from the sensor to DMT, the GeC_19_ sensor shows significant electronic changes and electron transfer from DMT to the sensor, especially in conformer 5.

**Fig. 6:**
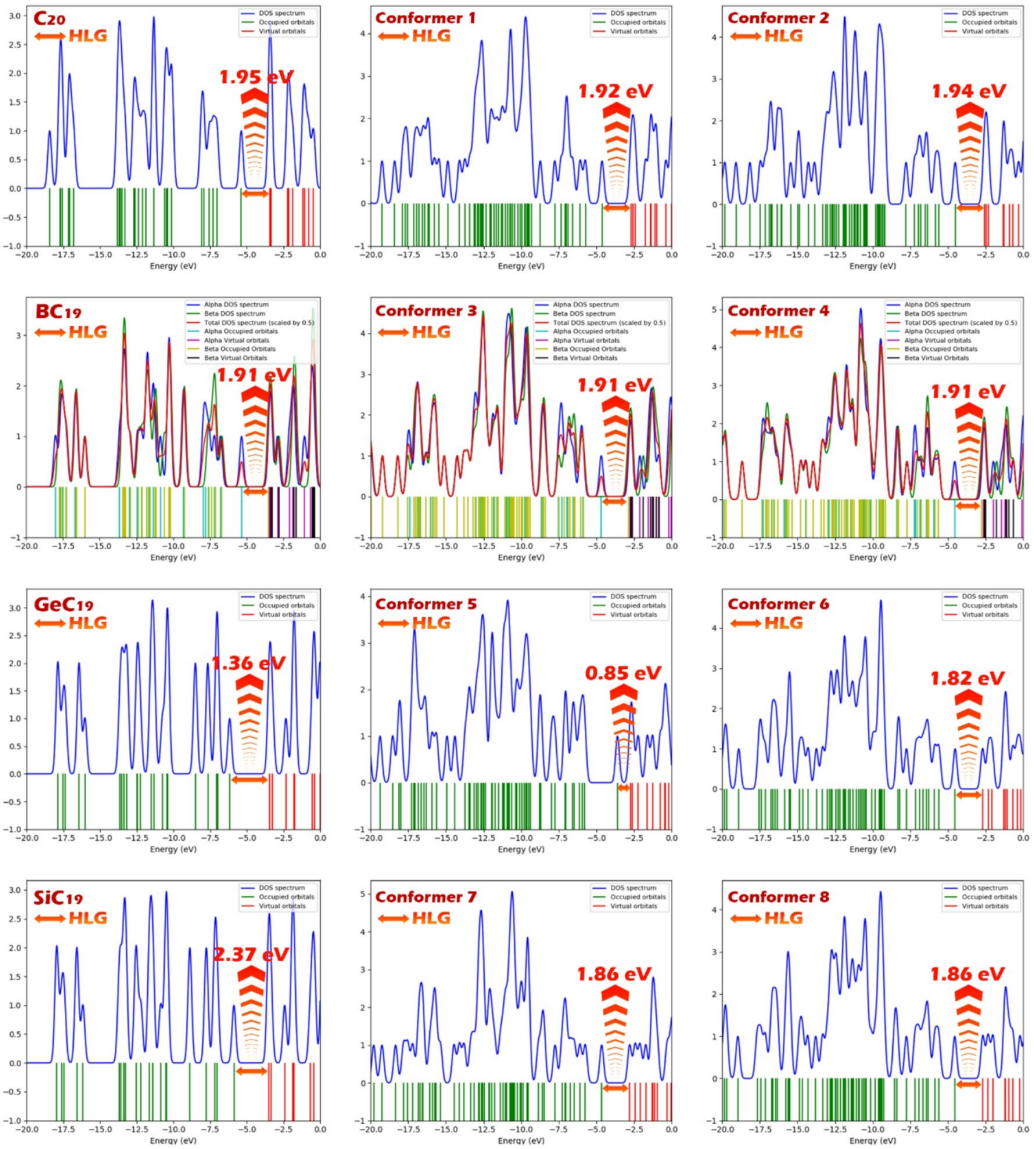
Density of states (DOS) spectra and corresponding HOMO-LUMO gaps (HLG) for C_20_, BC_19_, GeC_19_, SiC_19_ clusters and their DMT complexes (Conformers 1–8). The HLG values range from 0.85 eV to 2.37 eV, showing how interaction with DMT modifies the electronic structure and reduces the energy gap in certain conformers, particularly for GeC_19_@DMT.

Density of States (DOS) plot are an effective visual tool for illustrating the distribution of electronic states across energy levels, particularly around the Fermi level. One of their key advantages is the clear depiction of the energy gap between the HOMO and the LUMO. The region with no states between the HOMO and LUMO peaks directly corresponds to the HOMO-LUMO energy gap, offering a quick and intuitive way to assess the electronic characteristics of a molecule or material (Fig. 6)^70,71^.

In this study, the DOS plot validates the numerical data presented in Table 2. The positions of the HOMO and LUMO peaks in the DOS plots align well with the calculated orbital energies, and the visually observed energy gaps are consistent with the HLG values reported. This agreement between the DOS profiles and the tabulated results reinforces the reliability of the electronic structure calculations and confirms the changes in electronic properties upon complex formation with DMT.

**Fig. 7:**
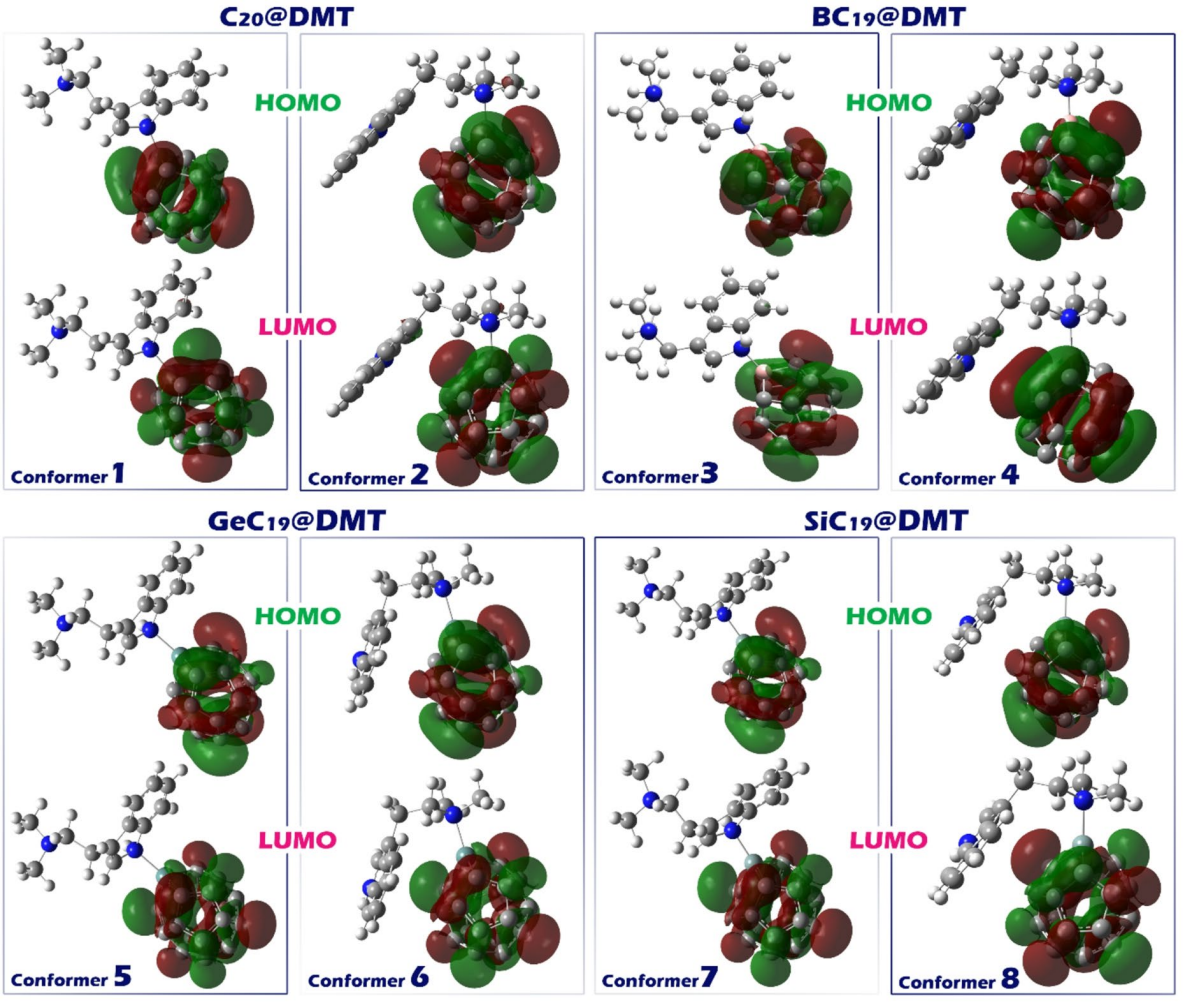
Frontier molecular orbitals (HOMO and LUMO) of the DMT complexes with C_20_, BC_19_, GeC_19_, and SiC_19_ clusters (Conformers 1–8). The green and red regions represent the positive and negative phases of the molecular orbitals, respectively. The orbital distributions illustrate the charge transfer interactions between DMT and the doped carbon clusters, highlighting variations in electronic coupling among different conformers.

Examining the spatial distribution of HOMO and LUMO orbitals provides important insights into how and where electronic interactions, such as charge transfer during sensing processes, occur (Fig. 7)^72^.

In all the conformers studied (C_20_@DMT, BC_19_@DMT, SiC_19_@DMT and GeC_19_@DMT), the HOMO and LUMO orbitals are located on the sensor. Having both HOMO and LUMO on the sensor indicates that the molecule can effectively interact with external stimuli (e.g., light, other molecules) because both electron donation and acceptance can occur at the same location. This can increase the responsiveness of the sensor to changes in its environment and make it more sensitive to the presence of analytes. The proximity of both orbitals on the sensor also increases the efficiency of charge transfer. This type of distribution maximizes the performance of the sensor, allowing it to interact effectively with its environment while maintaining stability and high performance.

#### Dipole moment

**Fig. 8:**
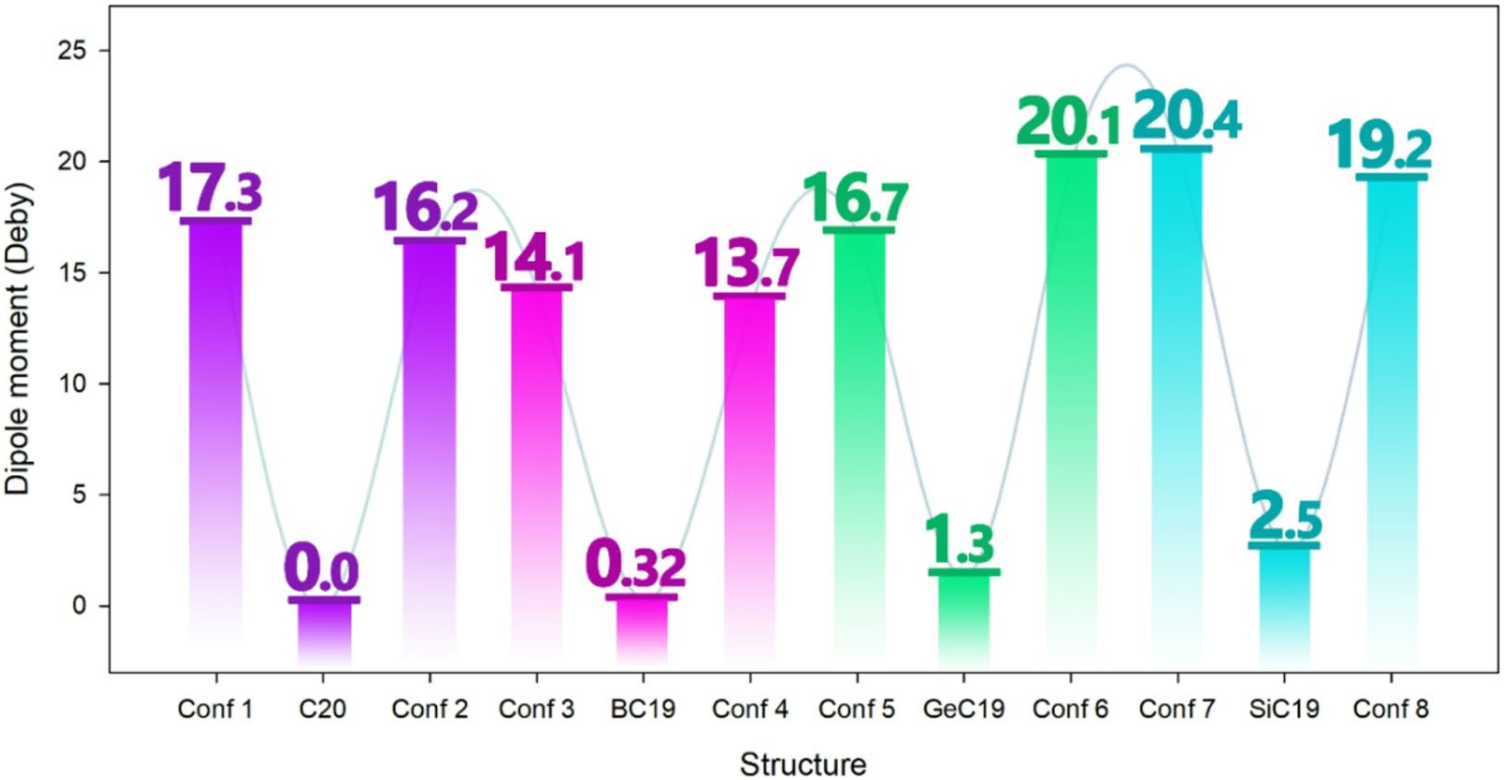
Dipole moments of pristine clusters (C_20_, BC_19_, GeC_19_, SiC_19_) and their DMT complexes (Conformers 1–8). The interaction with DMT significantly increases the dipole moment compared to the isolated clusters, with the highest values observed for GeC_19_@DMT (20.4 D) and SiC_19_@DMT (19.2 D), indicating strong charge polarization upon complex formation.

The dipole moment is a crucial parameter because it reflects the overall polarity of a molecule, which directly influences its solubility in polar solvents like water. Molecules with higher dipole moments tend to be more soluble in such solvents due to stronger intermolecular interactions like hydrogen bonding and dipole-dipole attractions^73,74^. Additionally, the dipole moment plays a significant role in generating electrical signals in sensing applications. Changes in the dipole moment upon interaction with target molecules can alter the local electric field and charge distribution, producing measurable electrical responses^75,76^. Therefore, the dipole moment was calculated for each of the designed structures, and the results are reported in Fig. 8.

The pristine sensors (C_20_, BC_19_, GeC_19_, and SiC_19_) exhibit very low dipole moments, with C_20_ showing a value of 0.0 Debye, and BC_19_ only slightly polar at 0.32 Debye. GeC_19_ and SiC_19_ display slightly higher dipole moments of 1.34 and 2.50 Debye, respectively, suggesting limited polarity in their unbound states.

Upon interaction with DMT, all sensor-DMT complexes (Conf 1 to Conf 8) demonstrate a dramatic increase in dipole moment. For example, the dipole moment of the C_20_@DMT complexes (Confs 1 and 2) rises sharply to 17.3 and 16.2 Debye, respectively, while BC₁₉@DMT (Confs 3 and 4) reaches 14.1 and 13.7 Debye. Similarly, GeC_19_@DMT (Confs 5 and 6) show increases to 16.7and 20.1 Debye, and SiC_19_@DMT (Confs 7 and 8) peak at 20.4 and 19.2 Debye.

The significant increase in dipole moment after DMT adsorption indicates increased polarity and consequently improved solubility of the complexes in polar solvents, which could facilitate better dispersion and interaction in biological or aqueous environments. Also, the significant dipole change upon DMT binding may lead to strong modulation of the local electric field, making these complexes highly responsive in electronic sensing applications. Measurable dipole shifts can contribute to detectable electrical signals.

### Sensor mechanism

#### Adsorption energy, recovery time and electrical conductivity

Adsorption energy, recovery time, and electrical conductivity are key parameters for evaluating the sensing performance of a material. Adsorption energy indicates how strongly the target molecule binds to the sensor (moderate values ensure stable yet reversible interactions)^77^. Recovery time, which depends on adsorption strength, reflects how quickly the sensor returns to its original state after detection, affecting its reusability^78^. Electrical conductivity determines the sensor’s ability to convert molecular interactions into measurable signals, directly influencing its sensitivity^79^. Each of these parameters was studied computationally, and the results are reported in Table 3.

**Table 3:** Adsorption energy (E_ads_), electrical conductivity ($\:\varvec{\upsigma\:}$) and recovery time ($\:\varvec{\uptau\:}$) values ​​in each of the studied structures.

Structure	Conformer	E_ads_ (kcal.mol^− 1^)	$\:\varvec{\tau\:}$ (s)	($\:\varvec{\sigma\:}$) (S/m)
C_20_	**-**	**-**	**-**	8.3 × 10^− 8^
BC_19_	**-**	**-**	**-**	2.4 × 10^− 7^
GeC_19_	**-**	**-**	**-**	3.4 × 10^− 15^
SiC_19_	**-**	**-**	**-**	2.7 × 10^− 11^
C_20_@DMT	1	−0.62	2.85 × 10^− 12^	1.6 × 10^− 7^
	2	−20.08	5.83 × 10^2^	1.2 × 10^− 7^
BC_19_@DMT	3	−23.53	1.83 × 10^5^	2.4 × 10^− 7^
	4	−40.78	1.18 × 10^30^	2.4 × 10^− 7^
GeC_19_@DMT	5	−25.75	7.79 × 10^6^	1.9 × 10^2^
	6	−25.72	7.5 × 10^6^	1.0 × 10^− 6^
SiC_19_@DMT	7	−0.63	2.90 × 10^− 12^	5.6 × 10^− 7^
	8	−18.82	6.37 × 10^1^	5.6 × 10^− 7^

The most stable conformer in each complex can be determined by the adsorption energies, which indicates that the more negative Eads is the more stable conformer. For C_20_@DMT, the more stable conformer is number 2 with Eads = −20.08 kcal.mol^− 1^ (versus − 0.62 for conformer 1); for BC_19_@DMT, the most stable conformer is number 4 with Eads = −40.78 kcal·mol − 1 (versus − 23.53 for conformer 3); for GeC_19_@DMT, the more stable conformer is number 5 with Eads = −25.75 kcal.mol^− 1^ (very slightly more stable than conformer 6 at −25.72); for SiC_19_@DMT, the most stable conformer is number 8 with Eads = −18.82 kcal.mol^− 1^ (versus − 0.63 for conformer 7). By looking at recovery time at these more stable conformers, τ now begins to lengthen to 5.83 × 10^2^ seconds for C_20_@DMT conformer 2 and 6.37 × 10^1^ seconds for SiC_19_@DMT conformer 8. GeC_19_@DMT shows very long values of τ in both conformers 5 and 6 of 7.79 × 10^6^ seconds and 7.5 × 10^6^ seconds (with a characteristic value of on the order of months). Contrastingly, BC_19_@DMT shows τ = 1.83 × 10^5^ seconds for conformer 3, and as expected, shows a significantly large τ = 1.18 × 10^30^ seconds for conformer 4, indicating virtually irreversible binding, which would be ideal for the capture/removal aspects of binding. Finally, the conductivity of the pristine supports is as follows: σ = 8.3 × 10^− 8^ S.m^− 1^ C_20_, σ = 2.4 × 10^− 7^ S.m^− 1^ BC19, σ = 3.4 × 10^− 15^ S.m^− 1^ GeC_19_, and σ = 2.7 × 10^− 11^ S.m^− 1^ SiC_19_. Following the adsorption of DMT, C20 shows a slight increase to σ = 1.6 × 10^− 7^ or 1.2 × 10^− 7^ S.m^− 1^ (conformers 1 and 2, respectively). BC_19_ does not show a notable difference and remains σ = 2.4 × 10^− 7^ S.m^− 1^ for both conformers. GeC_19_ has an exceptionally large dynamic range, with σ = 1.9 × 102 S.m^− 1^ for conformer 5 and 1.0 × 10^− 6^ S.m^− 1^ for conformer 6 (about a change of 10^16^ − 10^17^× from pristine for conformer 5). SiC_19_ reads at σ = 5.6 × 10^− 7^ S.m^− 1^ for both conformers (well below GeC_19_’s high and approximately the same low 10^− 7^ S.m^− 1^ range as for BC_19_). These changes suggest that GeC_19_ is the recommended candidate for a disposable electrochemical sensor for DMT, as it provides a much larger range of change in electrical conductivity post adsorption alongside a higher adsorption energy than the SiC_19_ solution (Eads = −25.75 and − 18.82 kcal.mol^− 1^ for the most stable conformers). For capture and removal, BC_19_ and SiC_19_ show the greatest potential as adsorbent materials; BC_19_ provides very high adsorption energy (as high as −40.78 kcal.mol^− 1^) while showing little to no perturbation of σ and effectively irreversible τ, while SiC_19_ provides a high enough adsorption energy (−18.82 kcal.mol^− 1^) for the most stable conformer combined with low absolute electrical conductivity with DMT present (σ = 5.6 × 10^− 7^ S.m^− 1^) for both materials to be suitable detection adsorbents (minimal signal drift) and particularly stable for adsorption-driven removal of DMT.

#### UV-Vis spectrum

**Fig. 9:**
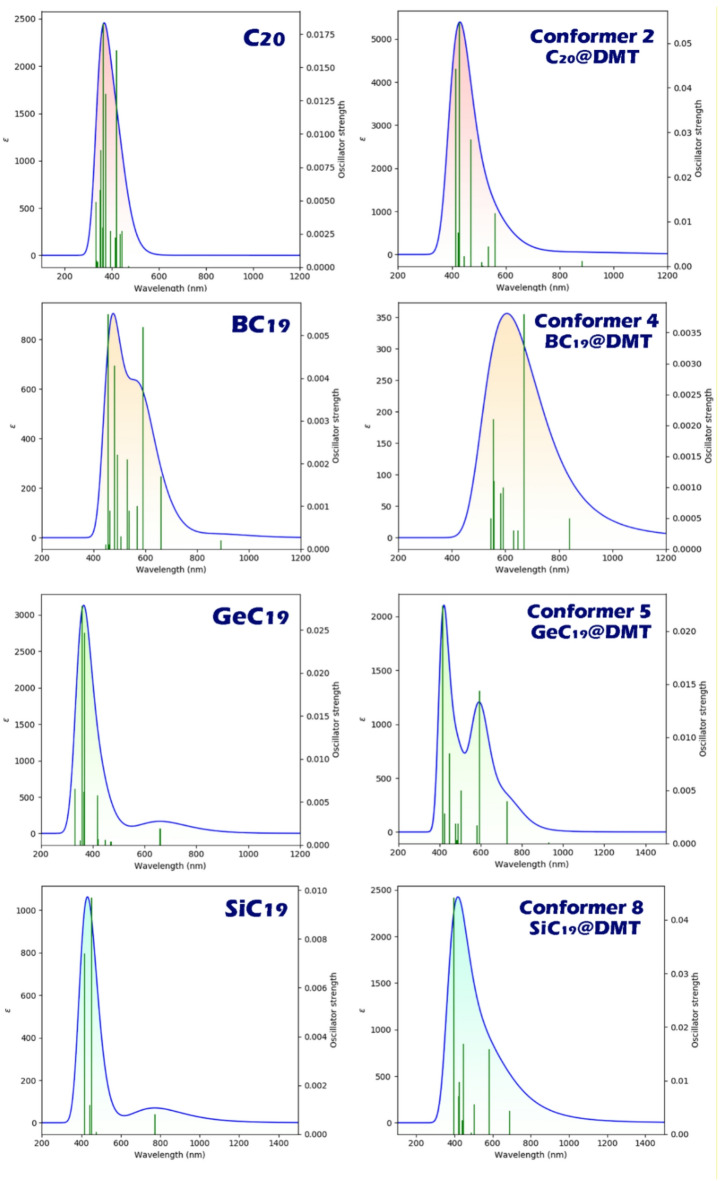
Simulated UV-Vis adsorption spectra of pristine clusters (C_20_, BC_19_, GeC_19_, SiC_19_) and their DMT complexes (Conformers 2, 4, 5, and 8). The plots show the adsorption coefficient (ε) and oscillator strength as functions of wavelength. Upon DMT interaction, notable redshifts and intensity variations are observed, indicating changes in electronic transitions and enhanced optical activity in the hybrid systems.

The maximum adsorption wavelength (λmax) and exciton energy (Eex) are important in electrochemical sensor design because they reflect how the sensor interacts with light and charge. The change in λmax after analyte binding indicates changes in the electronic structure of the sensor, which can be used for visual detection^80^. Lower exciton energy means easier charge separation, leading to better sensitivity and faster electronic response^81^. Each of these parameters (for the more stable conformers) was studied computationally to evaluate the capability of each of the designed structures as a colorimetric sensor. The results of these calculations are reported in Table 4; Fig. 9.

**Table 4:** Calculated values ​​of λmax (nm), and ex (eV) for each of the designed sensors in the presence/absence of DMT.

Structure	Conformer	λmax	Eex
C_20_	**-**	363	3.41
BC_19_	**-**	590	2.09
GeC_19_	**-**	359	3.44
SiC_19_	**-**	452	2.73
C_20_@DMT	2	428	2.89
BC_19_@DMT	4	670	1.84
GeC_19_@DMT	5	595	2.08
SiC_19_@DMT	8	398	3.11

The TD-DFT data in Table 4 indicate wavelength shifts upon DMT adsorption that are, in several cases, large enough to be experimentally distinguishable. Still, we agree that claims of straightforward “colorimetric sensing” should be moderated and framed against realistic spectral broadening and uncertainty. For pristine C_20_, λmax = 363 nm (Eex = 3.41 eV) red-shifts to 428 nm (2.89 eV) in C_20_@DMT, a Δλ of ~ 65 nm (ΔE ≈ 0.52 eV). For BC_19_, the 590 nm (2.09 eV) shift to 670 nm (1.84 eV) corresponds to ~ 80 nm (0.25 eV). GeC19 exhibits the largest bathochromic shift, from 359 nm (3.44 eV) to 595 nm (2.08 eV), i.e., ~ 236 nm (1.36 eV). In contrast, SiC19 shows a hypochromic shift from 452 nm (2.73 eV) to 398 nm (3.11 eV), ~ 54 nm (0.38 eV). Using a conservative Gaussian broadening on the order of 0.3 eV, the C_20_ (0.52 eV) and GeC_19_ (1.36 eV) shifts exceed a typical linewidth. They should be resolvable as a clear displacement of the adsorption envelope (even with modest instrumental resolution and matrix effects) provided oscillator strengths remain comparable. The BC_19_ shift (~ 0.25 eV) lies near a representative linewidth; it may still be observable as a change in the band maximum or via ratiometric two-wavelength readouts, but it will be more sensitive to solvent environment, baseline drift, and fitting choices. The SiC_19_ blue-shift (~ 0.38 eV) is also on the order of or slightly above a typical band width and should be experimentally detectable, though distinguishing a genuine hypochromic move from intensity redistribution among nearby transitions will require careful overlay analysis. Therefore, based on the results obtained, the calculated spectral perturbations strongly indicate that GeC_19_ (and to a somewhat lesser extent C_20_ and SiC_19_) could exhibit measurable adsorption-induced color/UV changes under experimental conditions, while BC_19_’s predicted red-shift, though consistent with adsorption, may be partially masked by line broadening and solvent/aggregation effects. In all cases, absolute TD-DFT excitation energies carry method-dependent errors (commonly a few tenths of an eV); thus, the most robust experimental strategy is to test for differential trends (pre/post-adsorption overlays with identical processing) and, where possible, use ratiometric or derivative spectroscopy to mitigate uncertainties in peak position and bandwidth.

#### NBO analysis

Studying NBO analysis and the second-order perturbation energy matrix (E^2^) in sensor-analyte complexes is essential for understanding the strength and nature of intermolecular interactions at the electronic level. NBO analysis reveals donor–acceptor interactions and electron delocalization, while E² quantifies the stabilization energy associated with these interactions. Higher E² values indicate stronger electron donation and greater stability of the complex, which enhances sensor sensitivity and selectivity. The main electronic transitions examined include σ → σ*, π → π*, lone pair (LP) → σ*, and LP → π* transitions. Among these, π → π* transitions are considered dominant in most sensor–analyte systems, especially those involving conjugated or aromatic systems, as they contribute most significantly to electronic excitation and spectral changes^82–85^.

**Table 5:** Calculated values ​​for the second-order perturbation energy matrix (E2) using NBO analysis in each of the conformers studied in this work.

Complex	Conformer	Donor (i)	Type	Acceptor (j)	Type	E^(2)^ kcal.mol^− 1^	E(j)-E(i) a.u.	F(i, j) a.u.
C_20_@DMT	2	C1-C2	$\:\sigma\:$	C1-C12	$\:{\sigma\:}^{*}$	3.24	1.23	0.056
		C4-C8	$\:\pi\:$	C7-C11	$\:{\pi\:}^{*}$	1.30	0.67	0.029
		N32	LP (1)	C30-C31	$\:{\pi\:}^{*}$	5.33	0.36	0.039
BC_19_@DMT	4	C1-C2	$\:\sigma\:$	C1-C12	$\:{\sigma\:}^{*}$	1.63	1.21	0.056
		C13-C14	$\:\pi\:$	C16-C17	$\:{\pi\:}^{*}$	10.67	0.33	0.076
		C4	LP (1)	C3-C6	$\:{\pi\:}^{*}$	26.07	0.17	0.101
GeC_19_@DMT	5	C1-C2	$\:\sigma\:$	C1-C12	$\:{\sigma\:}^{*}$	3.33	1.24	0.057
		C3-C6	$\:\pi\:$	C1-C12	$\:{\pi\:}^{*}$	20.02	0.35	0.077
		C8	LP (1)	C7-C11	$\:{\pi\:}^{*}$	50.77	0.15	0.096
SiC_19_@DMT	8	C1-C2	$\:\sigma\:$	C1-C12	$\:{\sigma\:}^{*}$	3.32	1.24	0.057
		C4-C5	$\:\pi\:$	C18-C19	$\:{\pi\:}^{*}$	1.65	0.31	0.021
		C8	LP (1)	C18-C19	$\:{\pi\:}^{*}$	32.93	0.14	0.071

For C_20_@DMT (Conformer 2), the NBO analysis reveals moderate stabilization energies (E^2^) for various transitions. The σ → σ* interaction between C1-C2 and C1-C12 (3.24 kcal/mol) and the π → π* transition involving C4-C8 and C7-C11 (1.30 kcal/mol) indicate weak to moderate electronic delocalization. The most significant interaction is the lone pair (LP) donation from N32 to the π* orbital of C30-C31 (5.33 kcal/mol), which suggests a degree of charge transfer that could facilitate electrochemical sensing. However, the overall E^2^ values are relatively low, implying limited sensitivity for colorimetric applications (see Table 5).

In BC_19_@DMT (Conformer 4), the NBO results highlight stronger interactions, particularly the π → π* transition between C13-C14 and C16-C17 (10.67 kcal/mol) and the LP (1) donation from C4 to the π* orbital of C3-C6 (26.07 kcal/mol). These high E^2^ values indicate significant charge transfer and electronic delocalization, which are essential for both colorimetric and electrochemical sensing. The strong LP → π* interaction suggests that BC_19_@DMT could exhibit pronounced optical changes (colorimetric response) and enhanced electrical conductivity upon DMT binding, making it a promising candidate for dual-mode sensing.

GeC_19_@DMT (Conformer 5) demonstrates even more robust interactions, with a notable π → π* transition between C3-C6 and C1-C12 (20.02 kcal/mol) and an exceptionally strong LP (1) → π* interaction involving C8 and C7-C11 (50.77 kcal/mol). These high E^2^ values reflect intense charge transfer and electronic redistribution, which are highly favorable for electrochemical sensing due to improved conductivity. The substantial LP → π* interaction also suggests significant optical changes, supporting its potential as a colorimetric sensor with high sensitivity.

For SiC_19_@DMT (Conformer 8), the NBO analysis shows a strong LP (1) → π* interaction between C8 and C18-C19 (32.93 kcal/mol), alongside weaker σ → σ* and π → π* transitions. These results severely limit its performance.

The NBO analysis underscores the superior sensor capabilities of BC_19_@DMT and GeC_19_@DMT, with their high E^2^ values and strong charge transfer interactions making them ideal for both colorimetric and electrochemical detection of DMT.

### NCI analysis

**Fig. 10:**
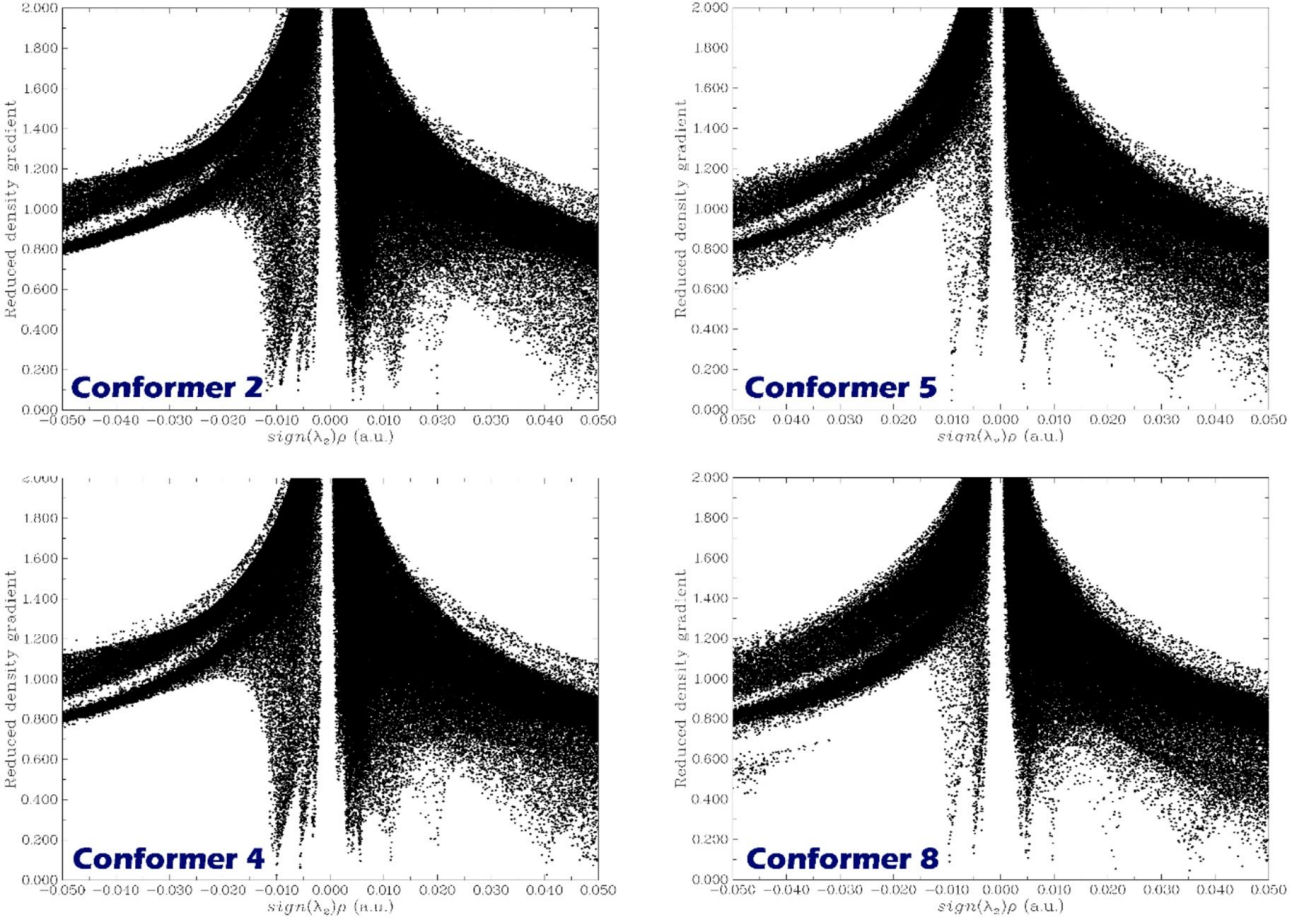
Reduced density gradient (RDG) vs. sign(λ_2_)ρ plots for selected DMT complex conformers (2, 4, 5, and 8). These plots reveal the nature and strength of noncovalent interactions within each system. Peaks near zero indicate weak van der Waals interactions, while negative regions correspond to attractive interactions such as hydrogen bonding, highlighting the presence of noncovalent stabilization in the sensor-DMT complexes.

Noncovalent interaction analysis (NCI) plays an important role in the design of complexes, as it provides a detailed understanding of weak interactions (such as van der Waals forces, hydrogen bonding, and π-π stacking) that significantly affect the stability and sensing performance of sensor-analyte systems^86,87^. Although these interactions are individually weak, their collective effect determines the binding strength, spatial orientation, and responsiveness of the analyte on the sensor surface. NCI analysis, through computational approaches, enables the visualization and quantification of these interactions by evaluating key parameters such as the reduced density gradient (RDG), electron density (ρ), and the sign of the second eigenvalue of the electron density Hessian matrix [sign(λ_2_)ρ]^88,89^. These criteria allow for the distinction between attractive, repulsive, and dispersion forces in the complex. In this study, the NCI plots for each designed complex are shown in Fig. 10.

For Conformer 2, the plot displays a distinct, sharp peak near sign(_2_) ≈ 0, indicating a region of strong interaction. The distribution of points shows a high density near the peak, suggesting significant non-covalent interactions. The symmetry of the plot, with a relatively even spread on both sides of the peak, points to balanced interaction behavior in this conformer.

Conformer 5 shows a similar pattern to Conformer 2, with a strong peak around the origin. However, the peak appears slightly narrower, suggesting that the interaction is more localized compared to Conformer 2. This indicates a stronger or more concentrated non-covalent interaction in conformer 5.

For Conformer 4, the plot still displays a pronounced peak near the origin, but there is a more noticeable tail extending on one side of the peak. This suggests that Conformer 4 may have a broader range of interactions, or that the non-covalent interactions are more distributed, making the peak slightly asymmetric.

Lastly, Conformer 8 shows a wider peak, indicating a broader range of interactions compared to the other conformers. The contours extend further along the sign(λ_2_ρ) axis, suggesting that Conformer 8 has a more distributed interaction profile. This means weaker or more dispersed noncovalent interactions over a wider area of the conformer.

### QTAIM analysis

Quantum Theory of Atoms in Molecules (QTAIM) provides a powerful theoretical framework for understanding the nature of atomic interactions by analyzing the topology of electron density, ρ(r), within a molecule^90^. A central feature of this approach is the identification of bond critical points (BCPs), which occur along the bond path where the gradient of the electron density, ∇ρ(r), is zero. These points serve as reliable indicators of bonding interactions and enable a detailed classification of bond types based on associated topological parameters^91^.

**Fig. 11:**
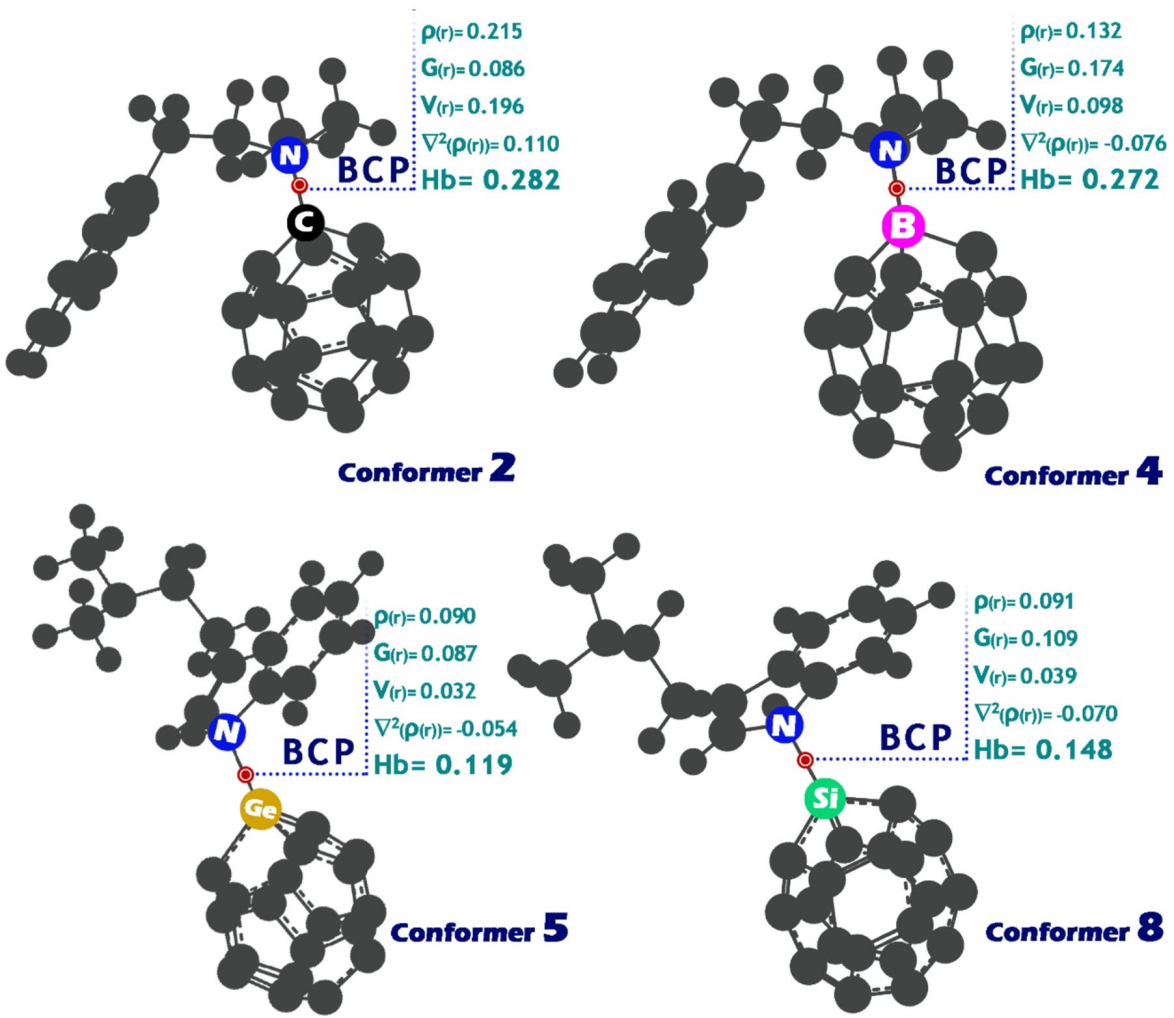
QTAIM results (total electron energy density (Hb), Laplacian of electron density (∇^2^ρ(r)), and kinetic energy density (G(r)) and potential (V(r))) for bond critical points (BCPs) in conformers 2, 4, 5, and 8.

Key properties evaluated at BCPs include the total electron energy density (Hb = G(r) + V(r)), which combines the kinetic energy density G(r) and potential energy density V(r), as well as the Laplacian of the electron density (∇^2^ρ(r))^92^. According to the widely accepted classification by Rozas et al., strong hydrogen bonds are indicated by negative values of both Hb and ∇^2^ρ(r), suggesting covalent-like character and high interaction strength. Medium-strength hydrogen bonds show Hb > 0 and ∇^2^ρ(r) < 0, reflecting dominant electrostatic interactions, while weak hydrogen bonds are characterized by Hb > 0 and ∇^2^ρ(r) > 0, typical of dispersive, van der Waals-type forces^93–95^. Accordingly, each of the values of ρ(r), V(r), G(r), and ∇^2^ρ(r) in BCP was calculated for each of the selected conformers, and the results were reported in Fig. 11.

In Conformer 2, the values of Hb = 0.282 and ∇^2^ρ(r) = 0.110 indicate that the interaction is a weak hydrogen bond. According to the Rosas classification, this suggests that the bond is of a dispersive, van der Waals-type character, which is consistent with Hb being positive and ∇^2^ρ(r) also being positive.

For Conformer 4, the Hb value of 0.272 combined with a negative Laplacian (∇^2^ρ(r) = −0.076) classifies this as a medium-strength hydrogen bond. This result suggests that the interaction is primarily electrostatic in nature, where Hb > 0 but the negative Laplacian indicates that the bond has some covalent-like character but is still dominated by electrostatic interactions.

Similarly, in Conformer 5, with Hb = 0.119 and ∇^2^ρ(r) = −0.054, the bond is also classified as a medium-strength hydrogen bond. Like Conformer 4, the positive value of Hb and the negative Laplacian indicate that the interaction is electrostatic, not as strong as a covalent bond but stronger than a purely van der Waals interaction.

Finally, Conformer 8 shows Hb = 0.148 and ∇^2^ρ(r) = −0.070, which also places it in the medium-strength hydrogen bond category. The bond in this conformer, like in Conformers 4 and 5, is driven by electrostatic interactions, as indicated by the positive Hb and negative Laplacian values.

### Comparison with other literature

While previous studies (Refs. 98–101) have demonstrated the potential of pristine and doped C_20_ fullerenes in detecting antidepressants, toxins, and volatile biomarkers, their applications have remained confined to relatively common chemical and biomedical targets. In contrast, the present work unveils (for the first time) the capability of C_20_-based nanostructures to detect Dimethyltryptamine (DMT), a psychoactive compound of considerable forensic and clinical relevance. This represents a novel application domain for C_20_, extending its utility from conventional chemical sensing to the selective and reusable detection of psychoactive drugs. The introduction of heteroatom doping (B, Si, Ge) not only enhances adsorption and conductivity properties but also demonstrates that C_20_ can be engineered for high-performance molecular recognition in complex biological and environmental matrices (See Table 6.).

**Table 6:** Comparative summary of adsorption and recovery characteristics of pristine and doped C₂₀ fullerenes for various molecular targets (Refs. 98–101) and this work.

Study/Reference	Target Molecule	Sensor Material	Method/Level of Theory	Eads	Recovery Time (τ)	Key Findings	Remarks
Jalali Sarvestani & Doroudi^96^	Amitriptyline	C_20_	DFT (B3LYP/6-31G*)	−27.2 kJ.mol^− 1^	1.3 × 10^− 9^ s	Moderate physisorption; conductivity ↑post-adsorption	Validated pristine C_20_ for antidepressant detection
Ajdari & Vahed^97^	Nortriptyline	C_20_	DFT (B3LYP/6-31G*)	−28.4 kJ.mol^− 1^	1.1 × 10^− 9^ s	Slightly stronger adsorption than amitriptyline	Demonstrated molecular selectivity
Ejaz et al^98^.	Ethyl butyrate	Fe-, Ni-, Cu-doped C_20_	DFT (PBE/def2-TZVP)	−34.5 – −48.0 kJ.mol^− 1^	10^− 10^ – 10^− 12^ s	Chemisorption at metal sites	Doping enhanced sensitivity
Khalaj Zeighami & Abdolkhani^99^	Aflatoxin M1	C_20_ and B-/N-doped	DFT (B3LYP/6-31G*)	−22.3 – −38.1 kJ.mol^− 1^	10^− 14^ – 10^− 15^ s	Stronger binding with B-doped; large dipole change	Fullerene efficient for toxin sensing
This work	DMT	Pristine C_20_, B-, Si-, and Ge-doped C_20_	DFT (B3LYP-D3/6-311G(d, p), CPCM water)	−0.62 to −40.78 kcal.mol^− 1^ (≈ −2.6 to −170.7 kJ.mol^− 1^)	2.85 × 10^− 12^ to 1.18 × 10^30^ s	BC_19_@DMT (Conf. 4) shows strongest binding (−40.78 kcal.mol^− 1^) but very slow recovery (τ = 1.18 × 10^30^ s); GeC_19_@DMT (Confs. 5–6) exhibits balanced adsorption (≈ −25.7 kcal.mol^− 1^) and fast recovery (τ ≈ 7.5 × 10^6^ s); both show strong dipole increase (up to 20.4 D) and conductivity improvement (up to 1.9 × 10^2^ S.m^− 1^)	GeC_19_@DMT identified as most promising sensor with optimal Eads, conductivity, and reusability for DMT detection.

### Future outlook

The promising results of this computational study on pristine and doped C_20_ fullerenes for DMT detection open several exciting avenues for future research and technological development. The next critical step involves experimental validation, particularly focusing on the synthesis and testing of boron-doped (BC_19_) and germanium-doped (GeC_19_) C_20_ fullerenes, which demonstrated superior adsorption energy, electrical conductivity, and optical responses in our simulations. Successful experimental confirmation would pave the way for integrating these nanomaterials into portable, low-cost sensor devices capable of real-time DMT detection in forensic, clinical, and environmental settings. Such devices could significantly enhance rapid on-site screening capabilities, addressing current limitations of conventional analytical techniques. Beyond DMT detection, the versatility of these doped fullerenes suggests potential applications in detecting other psychoactive substances or hazardous chemicals. In addition to sensing applications, these nanomaterials hold promise for biomedical uses, such as drug delivery systems for neurological disorders, given their ability to interact with biomolecules like DMT. Environmental monitoring represents another important application, where these sensors could be deployed for real-time detection of DMT and related compounds in water supplies or air, helping to mitigate public health risks associated with illicit drug contamination.

By pursuing these research directions, C_20_-based sensors could revolutionize detection technologies, offering rapid, reliable, and scalable solutions that benefit public health, safety, and environmental monitoring. The integration of computational insights with experimental validation will be crucial for unlocking the full potential of these innovative nanomaterials and translating them into practical, real-world applications.

## Conclusion

This thorough DFT assessment studied the use of pristine and doped C_20_ fullerenes (B-, Ge-, and Si-doped) as high-performance nanosensors and nanosorbents for rapid detection and capture of Dimethyltryptamine (DMT). The computational study generated significant results in the structural, electronic, and sensing properties, providing unique and highly advantageous features for each nanomaterial.

The adsorption energy (Eads) and electronic charge transfer values, derived from ECT and NBO analysis, were marked as the key contributors to sensor performance. The GeC_19_@DMT complex (Conformer 5) had the highest potential for use in disposable electrochemical sensors. It had a high adsorption energy of −25.75 kcal.mol^− 1^ capable of effectively capturing DMT with a vastly improved electrical conductivity (σ = 1.9 × 10^2^ S.m^− 1^) (more than 10^17^ times higher than pristine GeC_19_). The extraordinarily large increase in conductivity was the result of a change in the HOMO-LUMO gap (2.72 eV to 0.85 eV) and significant electron transfer from DMT to the sensor (ECT = −3.91). This change is substantial enough to generate a reliable and measurable electrical signal that can be used as an easily measured operational output for an electrochemical sensor.

On the other hand, BC_19_@DMT complex (Conformer 4) and SiC_19_@DMT (Conformer 8) were shown to be preferable adsorbents for the adsorption and removal of DMT. BC19 had the highest binding energy and essentially irreversible (Eads = −40.78 kcal.mol^− 1^, τ ≈ 1.18 × 10^30^ s), allowing for permanent capture. SiC_19_ had strong adsorption (−18.82 kcal.mol^− 1^) with a moderate recovery time, which is acceptable for both capture and a potential regeneration studies. The noteworthy increase in dipole moment upon binding DMT for all the complexes (up to 20.4 D) also indicates enhanced solubility in polar solvents, which is beneficial for their dispersions in aqueous or biological systems.

The UV-Vis analysis suggested a significant bathochromic shifts, especially in the case of GeC_19_@DMT (Δλ ≈ 236 nm), indicating the possibility of colorimetric detection. In addition, the QTAIM and NCI analyses also provided evidence of stabilizing non-covalent interactions, namely hydrogen bonds (ultimately calculate as medium-strength) and van der Waals forces, that provide evidence of the observed adsorption strengths and selectivity.

It is important to note that these are compelling, but still results from computational modelling, and further confirmation and application to devices will require the successful synthesis of these doped fullerene nanostructures and experimental verification. We hope that this work will inspire future experimental research to investigate these new, efficient, ready-to-use devices for DMT detection and removal.

## Data Availability

All data generated or analyzed during this study are included in this published article.
